# Effects of element complexes containing Fe, Zn and Mn on artificial morel’s biological characteristics and soil bacterial community structures

**DOI:** 10.1371/journal.pone.0174618

**Published:** 2017-03-28

**Authors:** Qingya Liu, Huimei Liu, Ciqiong Chen, Jinmei Wang, Yu Han, Zhangfu Long

**Affiliations:** 1 Key Laboratory of Bio-resources and Eco-environment (Ministry of Education), College of Life Sciences, Sichuan University, Chengdu, P.R. China; 2 Sichuan Tongfeng Science & Technology Co. Ltd, Chengdu, P.R. China; National Centre For Cell Science, INDIA

## Abstract

This study described the effects of elements (including Fe, Zn, Mn and their complexes) on the following factors in artificial morel cultivation: the characteristics of mycelia and sclerotia, soil bacterial community structures, yields and contents of microelements. The results indicated that the groups containing Mn significantly promoted mycelia growth rates, and all the experimental groups resulted in higher yields than the control (P<0.01), although their mycelia and sclerotia did not show obvious differences. It was also found that *Proteobacteria*, *Chloroflexi*, *Bacteroides*, *Firmicutes*, *Actinobacteria*, *Acidobacteria* and *Nitrospirae* were the dominated bacterial phyla. The Zn·Fe group had an unexpectedly high proportion (75.49%) of *Proteobacteria* during the primordial differentiation stage, while *Pseudomonas* also occupied a high proportion (5.52%) in this group. These results suggested that different trace elements clearly affected morel yields and soil bacterial community structures, particularly due to the high proportions of *Pseudomonas* during the primordial differentiation stage.

## Introduction

*Morchella spp* (morel) is highly prized for its medicinal and nutritional qualities, such as antioxidant, anti-inflammatory and antitumor activities, as well as strengthening the immune response [1~^4^]. According to previous experiments, morel mycelia growth and fruit-body (ascocarp) formation are strongly affected by many factors, such as temperature, humidity, illumination, air, pH and nutrition. The first indoor cultivation of morels was reported by Ower [5–6], but a great breakthrough had not been achieved in large-scale application until 2010.

It is common practice to cover morels with soil during cultivation to support soil microbes that are responsible for the promotion of primordial differentiation and ascocarp growth. However, thus far, we have known very little about how soils affect the growth and development of morels. Nevertheless, it has been demonstrated that soil microbes are important components of soil ecosystems and that bacteria are widely distributed in soil [7–10]. Inorganic fertilizer is commonly used for farmland management and can affect the soil microbial community structure [11,12]. Trace elements are usually conducive to the metabolic efficacy of edible mushrooms, particularly Fe, Zn, Mn and Cu, and may significantly affect mushroom growth and development. It was found that artificial morel ascocarps are rich in Fe, Zn and Mn [13], while many edible fungi (including wild morels) can convert inorganic metals into organic metal compounds that can be easily utilized by human beings [14,15]. Each trace element has special physiological functions that can influence fungal growth rates and soil bacterial community structures [16,17].

A few studies have reported that trace elements found in fruit bodies differ by strain types and applied fertilizers [13,18,19]. Our previous studies also found that some trace elements (Fe, Zn and Cu) affected soil microflora, as well as mycelia growth rates and yields in morels [13,20]. However, little is known about how trace elements affect morel soil microbial community structures, and whether bacteria in the soil affect morel yield. This study primarily reports the effects of some trace elements (Fe, Zn, Mn and their complexes) on morel mycelia growth rates, yield and soil bacterial community structures. The objective of the study is to discover not only some relationships among trace elements, yield and bacteria but also whether trace elements or bacteria affect the development of morel.

## Materials and methods

### Strains and chemical agents

The strain used in this study was preserved at 4°C in the authors’ laboratory [20]. Zinc sulfate (ZnSO_4_∙7H_2_O), manganous sulfate (MnSO_4_∙4H_2_O), ferrous sulfate (FeSO_4_∙7H_2_O) and other chemical reagents were analytically pure and purchased from local chemical reagent companies.

### Laboratory tests comparing the differences in mycelia growth

The stock culture of the experimental strain, which was preserved in a slant tube, was activated on PDA Petri dishes (potato 200 g/L, glucose 20 g/L, agar 18 g/L) and incubated under 18°C for four days. The mycelia plugs (6 mm in diameter) were sub-cultured in PDA media, which were treated with three single elements (including ZnSO_4_ (Zn), FeSO_4_ (Fe), MnSO_4_ (Mn)), three binary elemental complexes (including Zn∙Fe, Fe∙Mn and Zn∙Mn; w/w = 1:1) and one ternary complex (Zn·Fe·Mn; w/w/w = 1:1:1). Their final contents were 100 mg/L, while pure PDA media was used as the control group. Each group was triplicated and incubated under 18°C for 21 days. Mycelia growth and sclerotia formation were observed using a zoom stereo microscope (Leica, Germany). Mycelia growth rates were calculated using the formula reported by Liu et al (2015) [13] and were measured once a day until the mycelia had grown to the edge of the plates.

### Outdoor planting experiment

The outdoor experimental field was in Longxing Town, Chongzhou, Sichuan, P.R. China, and each experimental plot was 10 m^2^ in size. The experimental strain was reactivated using PDA media and incubated under 16~18°C for 15 days and then was transferred into the newly sterile spawn media (packaged in a 450-mL bottle, containing 70% wheat grains, 15% sawdust, 10% chaff, 1% vermiculite and 4% pure soil at a natural pH value). After 15 days of cultivation under 18°C, there were many sclerotia adhering to the glass walls. Approximately one bottle of spawn per square meter (m^2^) were sowed in the experimental plots and covered with fine soil (3~5 cm in depth) to support the subsequent production of morels. It was necessary to build a sunshading net (2 meters above the ground) prior to seeding, and water mist was thoroughly sprinkled on the soil until two or three days after sowing when the soil moisture content reached 22%. About twenty days later, the frost-like sclerotia of the morels were covering the soil surface. The mycelia and sclerotia were observed by collecting the frost-like soil blocks and using a zoom stereo microscope (Leica, Germany). After the collection, nutritional bags containing the same solid spawn media were added to the soil (approximately 3 bags per square meter) to rejuvenate the mycelia. There were seven experimental groups of different elements, including three single elements (6 g/m^2^ of each element), three binary elemental complexes (3 g/m^2^ of each element) and a ternary elemental complex (2 g/m^2^ of the element). The trace elements were resolved in 5 L of pure water, with an equal volume of pure water used as the control. They were sprayed onto the soil surfaces one month after seeding. Soil samples were collected during the primordial differentiation stage and the ascocarp growth stage (approximately two and three months after seeding, respectively).

### Soil bacterial community structures analyzed using high-throughput sequencing

#### DNA extraction and 16S rRNA MiSeq sequencing

The soil genomic DNAs were extracted according to the previous report [21]. The universal primers 515F (5'-gtgccagcmgccgcggtaa-3') and 909R (5'-ccccgycaattcmtttragt-3') with a 12-nt unique barcode were used to amplify the V4 hyper variable regions of the 16S rRNA genes for pyrosequencing using a MiSeq sequencer [22,23]. Each sample was amplified in a 25 μL reaction and followed the program which reported by Tamaki et al [24]. Two PCR reactions were conducted for each sample, and their products were combined and subjected to electrophoresis using a 1.0% agarose gel. The bands with the correct size were separately excised and purified using the SanPrep DNA Gel Extraction Kit (Sangon Biotech, Shanghai, China). The products were quantified with a Nanodrop. All samples were pooled together at equal molar amounts. The sequencing samples were prepared using the TruSeq DNA kit (Illumina, Shanghai, China) according to manufacturer’s instructions. The purified library was diluted, denatured, re-diluted, and mixed with PhiX (equal to 30% of final DNA amount) according to the Illumina library preparation protocols. Then, the products were applied to an Illumina Miseq system for sequencing with 2×250 bp, using the Reagent Kit v2 (Illumina, Shanghai, China) as described in manufacturer’s manual.

#### Data analysis for bacteria community structures

The sequence data were processed using QIIME Pipeline–Version 1.7.0 (http://qiime.org/). All sequence reads were trimmed and assigned to each sample based on their barcodes. High quality sequences (length > 150 bp, without ambiguous base ‘N’ and an average base quality score > 30) were used for downstream analysis. The sequences were clustered into operational taxonomic units (OTUs) at a 97% identity threshold. The aligned ITS gene sequences were used for a chimera check using the Uchime algorithm [25]. All samples were randomly resampled to 6,449 reads. We conducted alpha-diversity (phylogenetic distance whole tree, chao1 estimator of richness, observed species and Shannon’s diversity index) and beta-diversity (PCoA, UniFrac) analyses, and the rarefaction curves were generated from the observed species. Taxonomy was assigned using the Ribosomal Database Project Classifier [26].

#### Sequence accession numbers

Sequence data have been deposited in the NCBI Sequence Read Archive (SRA) database with the accession number SRP093475.

### Mineral determination of the morel ascocarp

When the ascocarps were close to maturity, the morels were carefully collected until the end of March, and the yields of all the groups were measured in triplicate. Randomly chosen morel ascocarps from each group were prepared by the acid digestion method described in the literature [19], and the contents of five microelements (including zinc, ferric, manganese, copper and selenium) were measured by the standard procedure using an inductively coupled plasma atomic emission spectrophotometer (ICPQ-1000, Shimadzu, Japan) according to the method described in our previous report [13]. Yields and microelements of ascocarp were analyzed using SPSS (Version 17).

## Results

### The effects of trace elements on the mycelial growth rate and sclerotial formation

The growth rates of morel’s mycelia were clearly enhanced by the different elements (Table 1); in particular, the four groups that contained with Mn had greater growth rates than the control (19.87%~23.92%, P<0.05). Sclerotia were emerged first in Mn group (9 days after inoculation), and emerged last in Zn∙Mn group (16 days after inoculation). The order of the sclerotia emergence was: Mn>Zn>Zn∙Fe>Zn∙Fe∙Mn>Fe∙Mn>Fe>ck>Zn∙Mn.

**Table 1:** Effects of trace elements on mycelia growth and sclerotial formation.

Groups	Mycelia			Sclerotia		
	Growth rate(mm/d)	Vigor	Color	Earliest (d)	Color	Distribution
Zn	11.56±0.05^a^	+	white	10	yellow	dispersed
Fe	11.56±0.08^a^	+	white	14	white	dispersed
Mn	13.56±0.03^b^	+++	white	9	yellow	dispersed
Fe∙Zn	11.94±0.03^a^	++	light yellow	11	brown	centered
Fe∙Mn	13.33±0.06^b^	+++	white	13	white	centered
Zn∙Mn	13.78±0.01^b^	++++	white	16	white	centered
Fe∙Zn∙Mn	13.50±0.11^b^	+++	white	12	light yellow	dispersed
Ck	11.12±0.05^a^	+	white	15	white	dispersed

Superscript letters (a–b) indicate that the values of different fertilizers sharing a common letter were not significant at P < 0.05. “+,++,+++ and ++++” indicate the vigor of mycelia from weak to strong. The growth rate was the average value of the mycelia on the 2^nd^, 3^rd^ and 4^th^ day after inoculation. The color and dispersion degree of the sclerotia were observed on the 21^st^ day after inoculation.

### The morphology of mycelia and sclerotia under a zoom stereomicroscope

When mycelia covered approximately the whole plate (3 days after inoculation), the morphology of the mycelia were observed under a zoom stereomicroscope. The results (S1 Fig) demonstrated that the mycelia were wide, flat, and spirally twisted (approximately 12.5 μm), and no significant difference was found among the groups.

**Fig 1:**
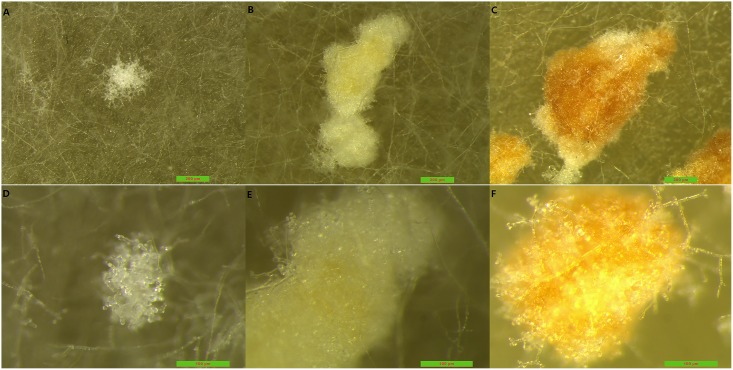
Process of sclerotia formation in PDA media under stereomicroscope. (A~ C) The sclerotia are in the early, middle and late stages, respectively (5x) and (D~F) represent the same stage under 16 ×; The color of sclerotia were from white to faint yellow and final in brown.

**Fig 2:**
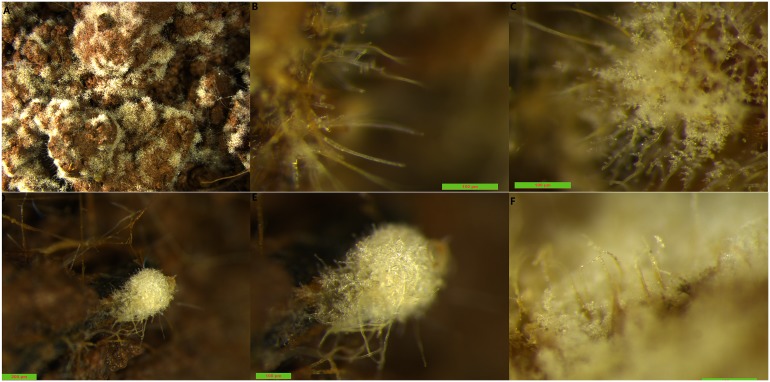
Morphologies of mycelia and sclerotia on the soil surface under stereomicroscope. (A~C) The morphology of mycelia under 1x, 16x, and 16x magnification of a zoom stereomicroscope, respectively; (D~F) The morphology of sclerotia under 5x, 10x, and 16x magnification of a zoom stereomicroscope, respectively. The morphology of mycelia and sclerotia between medium and soil showed significantly different.

It was discovered that many bridged mycelial branches (S2 Fig) were finely formed, then sclerotia began to originate from the aggregated bridges. The sclerotia were initially white and changed color from faint yellow to brown when they matured (Fig 1). The mycelia grown in the soil were short, erect, and tufted before the sclerotia (in the form of a white fungal cream) covered the soil surface (Fig 2). It was also observed under the zoom stereomicroscope that there were many short vertical mycelia on the sclerotia surface.

### Effects of trace elements on soil bacterial community structures

#### Rarefaction curves and diversity indices

The rarefaction curves were generated at a 97% identity threshold (S3 Fig), and the sequencing depth of samples from most groups was reached to a suitable degree. Bacterial sequences of morel-grown soils from experimental groups were also clustered into operational taxonomic units (OTUs) and other diversity indices (including Shannon and Chao1) were estimated (shown in S1 Table). The OTUs of most groups (except Zn∙Fe) were similar during the two sampling stages (2390.32 on average). The highest (6259.93) and lowest (2146.03) Chao1 indexes during primordial differentiation stage were found in groups with Fe and Zn∙Fe, respectively. The Chao1 indexes during the primordial differentiation stage (5413.37 on average) demonstrated the following trend: Fe>Fe∙Mn>Zn∙Mn>Zn>Mn>ck>Zn∙Fe∙Mn>Zn∙Fe. The Chao1 indexes of the single element and binary elements containing Mn were higher than that of ck. However, this trend was changed during the ascocarp growth stage; Chao1 indexes which higher than ck were found in experimental groups containing Zn, Mn or Fe∙Mn. The Shannon indexes (10.3 on averages) demonstrated the following trend during the primordial differentiation stage: Mn>Zn>Fe> Zn∙Mn>ck>Zn∙Fe∙Mn>Fe∙Mn>Zn∙Fe, while the highest (10.96) and the lowest (7.65) Shannon indexes were found in groups with Zn and Zn∙Fe.

#### Comparison of bacterial community structures at the phylum level

**Fig 3:**
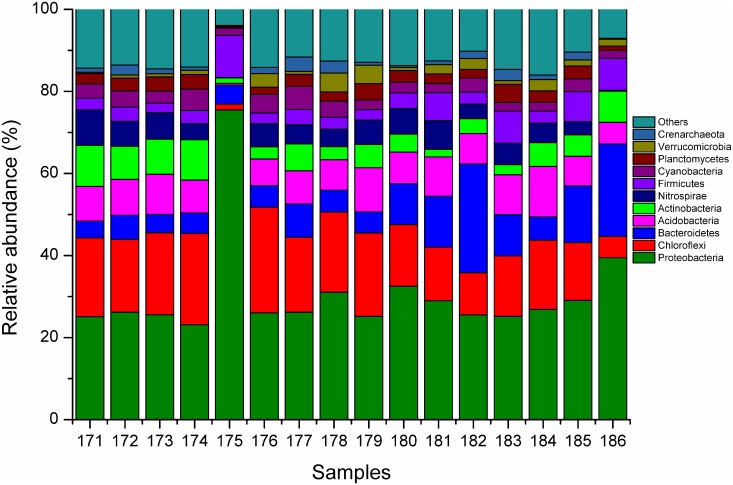
Histograms of soil bacteria richness. 571–578 represent the control (ck), Mn, Zn, Fe, Zn∙Fe, Fe∙Mn, Zn∙Mn and Zn∙Fe∙Mn groups, respectively, during the primordial differentiation stage. 579–586 represent the same groups of above during the ascocarp growth stage. The length of columns indicate the proportions of bacteria at the phylum level.

The bacterial community structure was noticeably affected by different trace elements (Fig 3). Seven dominant bacterial phyla, including *Proteobacteria*, *Chloroflexi*, *Bacteroidetes*, *Acidobacteria*, *Nitrospirae*, *Firmicutes* and *Actinobacteria*, were found in all groups, and their total percentages ranged from 74.72% to 93.63% (Table 2). The total bacterial percentage was similar across groups, except for the Zn∙Fe group during the primordial differentiation stage. However, the proportions of each bacteria phylum clearly differed across groups. *Proteobacteria* and *Chloroflexi* were the first and second most dominant bacterial phyla in most groups; the total proportion of these two bacterial phyla ranged from 35.81% (Fe group) to 76.92% (Zn∙Fe group).

**Table 2:** Proportions of predominant bacterial phyla in morel-grown soil.

Groups	Acidobacteria	Actinobacteria	Bacteroidetes	Chloroflexi	Firmicutes	Nitrospirae	Proteobacteria	Total
171(ck)	8.43	10.03	4.09	19.21	2.87	8.62	25.09	78.34
172(Mn)	8.74↑	8.08↓	5.92↑	17.74↓	3.51↑	6.06↓	26.17↑	76.22↓
173(Zn)	9.78↑	8.56↓	4.49↑	19.94↑	2.31↓	6.44↓	25.61↑	77.13↓
174(Fe)	8.02↓	9.84↓	5.03↑	22.22↑	3.18↑	3.93↓	23.14↓	75.36↓
175(Zn∙Fe)	0.39↓	1.35↓	4.61↑	1.43↓	10.24↑	0.12↓	75.49↑	93.63↑
176(Fe∙Mn)	6.52↓	2.93↓	5.28↑	25.75↑	2.55↓	5.69↓	26.00↑	74.72↓
177(Zn∙Mn)	8.06↓	6.54↓	8.14↑	18.28↓	3.68↑	4.74↓	26.17↑	75.61↓
178(Zn∙Fe∙Mn)	7.44↓	3.16↓	5.32↑	19.54↑	2.87-	4.28↓	31.07↑	73.68↓
179(ck)	10.78 ⇡	5.67 ⇣	5.13 ⇡	20.31 ⇡	2.58 ⇣	5.92⇣	25.19 ⇡	75.58 ⇣
180(Mn)	7.71↓ ⇣	4.36↓ ⇣	9.95↑ ⇡	15.04↓ ⇣	3.82↑ ⇡	6.27↑ ⇡	32.52↑ ⇡	79.67↑⇡
181(Zn)	9.60↓ ⇣	1.85↓ ⇣	12.40↑ ⇡	13.04↓ ⇣	6.83↑ ⇡	6.98↑ ⇡	28.99↑ ⇡	79.69↑⇡
182(Fe)	7.39↓ ⇣	3.63↓ ⇣	26.52↑ ⇡	10.26↓ ⇣	2.93↓ ⇣	3.55↓ ⇣	25.55↑ ⇡	79.83↑⇡
183(Zn∙Fe)	9.68↓ ⇡	2.49↓ ⇡	10.03↑ ⇡	14.75↓ ⇡	7.81↑ ⇣	5.21↓ ⇡	25.19- ⇣	75.16↓⇣
184(Fe∙Mn)	12.27↑ ⇡	5.82↑ ⇡	5.71↑ ⇡	16.86↓ ⇣	2.95↑ ⇡	4.74↓ ⇣	26.85↑ ⇡	75.20↓⇡
185(Zn∙Mn)	7.19↓ ⇣	5.28↓ ⇣	13.83↑ ⇡	14.08↓ ⇣	7.33↑ ⇡	3.18↓ ⇣	29.08↑ ⇡	76.97↑⇡
186(Zn∙Fe∙Mn)	5.23↓ ⇣	7.56↑ ⇡	22.55↑ ⇡	5.21↓ ⇣	7.79↑ ⇡	0.25↓ ⇣	39.46↑ ⇡	88.05↑⇡

The numbers 171~178 indicate the results for the experimental groups during the primordial differentiation stage, while 179–186 indicate those of the same group during the ascocarp growth period. Solid arrows indicate results of experiments compared with ck, while dashed arrows indicate results of each group compared by growth stage. Up or down arrows indicate a respectively higher or lower proportion than the compared group.

When the bacterial community structures of the experimental groups were compared with that of the control group during the primordial differentiation stage, *Proteobacteria* and *Bacteroidetes* proportions from most groups tended to be higher than those in ck, while *Actinobacteria*, *Nitrospirae*, and *Acidobacteria* were lower than ck. The highest proportion of *Proteobacteria* (32.34% on average) was found in the Zn∙Fe group (75.49%, 2.43~3.26 times higher than those of other groups), while the lowest proportion was found in the Fe group (23.14%). The highest proportion of *Bacteroidetes* (8.14%) was found in the Zn∙Mn group, while the lowest proportion was found in ck (4.09%); *Bacteroidetes* proportions from most experimental groups were higher (ranging from 9.78~99.02%) than that of ck during primordial differentiation stage. Alternatively, *Actinobacteria* and *Nitrospirae* proportions from all experimental groups were lower than ck, with the lowest proportions of these two phyla found in the Zn∙Fe group. Proportions of *Acidobacteria* in most groups (except Mn and Zn groups) were also lower than ck.

After comparing the proportions of predominant bacterial phyla in the primordial differentiation stage and the ascocarp growth stage, it was found that *Proteobacteria*, *Bacteroidetes* and *Firmicutes* increased for most groups during ascocarp stage, while *Acidobacteria*, *Actinobacteria*, *Chloroflexi* and *Nitrospirae* mostly decreased. The proportions of *Bacteroidetes* were rose across all groups during the ascocarp growth stage, an increase that ranged from 25.43% to 427.24% compared with that of primordial differentiation stage for each group. However, in the Zn∙Fe group, the proportions of *Acidobacteria*, *Actinobacteria*, *Bacteroidetes*, *Firmicutes* and *Chloroflexi* were higher in ascocarp stage than primordial differentiation stage, but the group in former stage produced lower proportions of *Nitrospirae* and *Proteobacteria*.

#### Bacterial community structure at genus level

Since the most predominant bacteria in the grown soil came from the *Proteobacteria* phylum, the associated bacterial genera that was found in all groups were specially selected to also be analyzed in this study (Table 3). Three genera, including *Rhodoplanes*, *Geobacter* and *Pseudomonas*, were most predominant, with the genus *Pseudomonas* having the highest proportions in most groups. During the primordial differentiation stage, there was the largest number of *Pseudomonas* in the control group and all the groups which contained Mn, but three single-element groups (Zn, Fe and Mn) had higher proportions of *Rhodoplanes* though other experimental groups (binary and ternary complexes) had lower proportions than the control group. The proportions of *Geobacter* and three unknown genera (belonging to *Comamonadaceae*, *Janthinobacterium* and *Syntrophobacteraceae*) were lower in the ck group than most experimental groups. The group with Zn∙Fe (No.175) had the highest proportion of *Acinetobacter* (23.36%), *Janthinobacterium* (8.18%), and *Pseudomonas* (5.52%) during primordial differentiation stage.

**Table 3:** Predominant genera of *Proteobacteria* in morel-grown soil.

Family; genus	Primordia differentiating stage								Ascocarp growth stage							
	171	172	173	174	175	176	177	178	179	180	181	182	183	184	185	186
Hyp; Rhodoplanes	1.64	2.16	2.18	1.87	0.06	0.64	1.48	0.79	1.45	1.29	0.91	0.71	0.75	1.66	1.04	0.91
Rho, unknown	0.58	0.64	0.77	0.75	0.02	0.54	0.73	0.48	0.85	0.60	0.52	0.48	1.00	0.73	0.54	0.31
Com; other	0.15	0.56	0.29	0.52	1.02	0.46	0.73	0.46	0.31	2.18	0.67	1.50	0.37	0.71	1.16	2.29
Jan; unknown	0.04	0.21	0.21	0.13	8.18	0.21	0.44	0.29	0.06	2.93	0.21	0.69	0.13	0.02	1.83	0.50
Geo;Geobacter	0.77	1.41	1.37	0.83	0.06	1.35	1.23	1.14	1.75	1.56	1.72	1.35	0.96	1.41	0.98	0.31
Hal; unknown	0.52	0.56	0.67	0.27	0.04	0.19	0.60	0.35	0.48	0.13	0.44	0.52	0.69	0.79	0.60	0.83
Myx;Anaeromyxobacter	0.94	0.98	0.42	0.29	0.04	0.62	0.49	0.29	0.46	0.56	1.04	0.31	0.56	0.42	0.37	0.40
Syn;unknown	0.71	1.41	1.21	0.73	0.17	0.94	0.83	0.77	1.18	1.66	1.04	0.75	1.02	1.33	0.75	0.15
Mor;Acinetobacter	0.08	0.06	0.04	0.02	23.36	0.08	0.02	0.02	0.02	0.19	0.44	0.02	0.46	0.08	0.35	4.59
Pse;Pseudomonas	2.47	0.50	0.67	1.43	5.52	2.51	2.10	4.22	1.101	1.81	5.23	1.79	1.12	0.58	2.83	4.15
Total	7.90	8.49	7.83	6.84	38.47	7.54	8.65	8.81	7.66	12.91	12.22	8.12	7.06	7.73	10.45	14.44

Hyp-Hyphomicrobiaceae; Rho-Rhodospirillaceae; Com-Comamonadaceae; Jan-Janthinobacterium; Geo-Geobacteraceae; Hal-Haliangiaceae; Myx-Myxococcaceae; Syn-Syntrophobacteraceae; Mor-Moraxellaceae; Pse-Pseudomonadaceae.

During the ascocarp stage, *Pseudomonas* was also the dominated genus in groups with Zn (5.23%), Zn∙Fe∙Mn (4.15%), Zn∙Mn (2.83%), Fe (1.79%) and Ze∙Fe (1.12%). However, the control group had the highest proportion of *Geobacter* (1.75%) and *Rhodoplanes* (1.45%) which was higher than that of most experimental groups (except Fe∙Mn group, No.184).

#### Principal Coordinates Analysis (PCoA)

Using the weighted UniFrac distance metric, PCoA showed that 175 (Zn) had the greatest diversity, followed by 186 (Zn∙Fe∙Mn) and 182 (Mn) (S4 Fig). The dispersion levels of the groups within the ascocarp growth stage were higher than those of the groups during the primordial differentiation stage. There was a definite change in experimental groups, particularly in groups with Fe, Zn∙Fe, and Zn∙Fe∙Mn, respectively. The relative degrees of variance in those groups were as follows: Zn∙Fe>Zn∙Fe∙Mn>Fe>Zn∙Mn>Zn>Mn>Fe∙Mn>ck.

### Yields and contents of microelements in morel ascocarp

**Fig 4:**
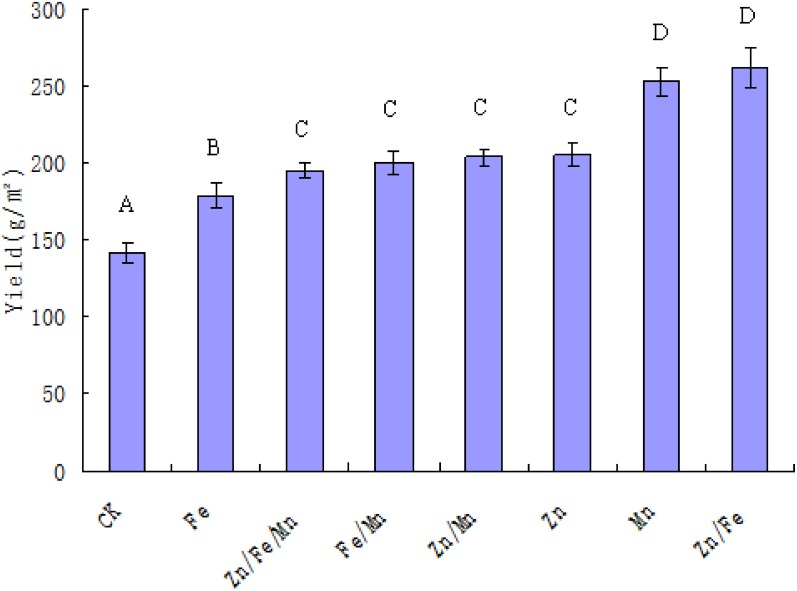
Morel yields of groups with different element treatments. Values sharing a common capitalized letter (A-D) were not significant at P<0.01.

The relative yields of artificial morel demonstrated the following trend: Zn∙Fe>Mn>Zn>Zn∙Mn>Fe∙Mn>Zn∙Fe∙Mn>Fe>ck (Fig 4). The yields from the groups that were sprayed with elements (including groups with Zn, Fe∙Mn, Zn∙Mn or Zn∙Fe∙Mn) were not significantly different, but the morel yields of all experimental groups were significantly higher than that of the control group (P<0.01). The contents of five microelements (including zinc, ferric, manganese, copper and selenium) were also determined (Table 4). Ferric content was the highest, while manganese and copper were the lowest mineral levels. The ascocarp with the highest content of selenium was found in the Zn∙Mn group, while zinc and ferric were lowest in the Zn∙Fe∙Mn group.

**Table 4:** Contents of microelements in artificial morel ascocarp (mg/kg dw).

Minerals	Group							
	ck	Mn	Zn	Fe	Zn∙Fe	Fe∙Mn	Zn∙Mn	Zn∙Fe∙Mn
Ferric (Fe)	529.00±3.4^a^	600.25±3.0^b^	399.00±1.56^c^	748.00±8.08^d^	652.25±3.1^e^	1247.5±17.64^f^	791.75±2.23^g^	379.32±2.53^c^
Zinc (Zn)	170.35±1.0^a^	205.90±0.9^b^	201.15±2.04^bc^	205.90±1.09^b^	199.13±3.05^c^	250.75±3.65^d^	225.71±0.75^e^	165.71±1.36^a^
Copper (Cu)	35.78±0.4^a^	51.43±2.2^b^	50.63±0.79^b^	38.00±0.27^a^	46.23±0.47^c^	50.05±1.08^b^	41.83±0.78^d^	49.32±0.8^b^
Manganese (Mn)	33.98±2.0^a^	49.63±0.5^b^	59.65±0.09^de^	38.48±0.92^c^	57.83±1.07^d^	62.13±1.03^e^	53.32±0.62^f^	51.65±0.65^bf^
Selenium (Se)	60.7±4.1^a^	92.03±0.9^b^	140.68±2.0^c^	92.03±1.68^b^	151.03±4.69^d^	161.38±2.43^e^	210.05±3.96^f^	42.69±0.72^g^

Values sharing a common superscript letter (a~g) were not significant at P < 0.05.

## Discussion

Thus far, large-scale planting of morels in China is usually field-based; therefore, yields are greatly affected by natural conditions and soil. Although some growers can cultivate morels indoors during the normal planting season, the production cost is still very high. Because morels must be planted in soil or covered with soil, it is very important to discover how the soil supports morel growth. It is well known that there are substantial amounts of microbes and nutrient elements in soil that are necessary for morel growth. Therefore, this study selected and applied those elements which were rich in the ascocarp of morel, such as Fe, Zn and Mn, to stimulate differences in morel growth and yield while comparing their effects on morel-grown soil community structures.

In this study, it was confirmed that there were not clear differences in mycelia characteristics among the control group and those treated with trace elements; they differed significantly in mycelia growth rates, yields and ascocarp mineral contents, especially in the Zn∙Mn group. The mycelia growth rates of experimental groups which contain Mn were higher than others; therefore, there was a synergetic effect between Zn and Mn. The earliest sclerotia, which is crucial to fruit body formation [5,6], first appeared in the Mn group, while Fe, Zn and their combination also had better effects on the formation of sclerotia. Those results suggested that a Zn∙Fe∙Mn complex is necessary for morel growth. However, some unexpected results of this experiment suggested that the three-element complex was not be prepared in the best proportions, so it is necessary to further study a suitable proportion for these three elements, even combined with other factors.

This study also demonstrated that artificial morel’s soil bacterial diversity and richness were significantly affected by different elements. Seven predominant phyla were found in all groups, including *Proteobacteria*, *Chloroflexi*, *Actinobacteria*, *Acidobacteria*, *Nitrospirae*, *Bacteroidetes* and *Firmicutes*. It is very interesting that *Proteobacteria* was found in the largest proportion at an unexpected level in the Zn∙Fe (75.49%, primordial differentiation stage) and Zn∙Fe∙Mn groups (39.46%, ascocarp growth stage), while the yields of these groups were also significantly higher than the control group. It has been reported that the secretions of some bacteria may promote morel growth [27], perhaps the increased *Proteobacteria* bacteria may have contributed to the improved ascocarp yield. Further analyses verified that *Pseudomonas*, *Geobacter* and *Rhodoplanes* were the main bacteria in *Proteobacteria*. We know that some strains of *Pseudomonas* had bio-control properties, such as killing parasitic fungi and some phytophagous nematodes [28], as well as stimulating hyphal growth, reducing ethylene production and inducing primordium formation of *Agaricus bisporus* [29]. However, *Pseudomonas* was the most common bacteria (5.52%) in the Zn∙Fe group (primordial differentiation stage), and this group also harvested the highest yield of ascocarps. Hence, we hypothesize that *Pseudomonas* may have such effect on morel primordial differentiation, which is the key to the formation of the fruit body throughout the morel life cycle [30]. On the other hand, *Geobacter* is usually associated with electron transport and conducive to oxidizing organic substrates [31], while *Rhodoplanes* was observed using organic compounds such as carbon sources and electron donors [32]. These bacteria could help organic compounds converted to carbon sources which can be utilized by morels.

*Nitrospirae* plays a key role in the nitrogen cycle [33], its nitrification supplies a nitrogen source for morel growth. It was lower than ck in most experimental groups, perhaps due to a decrease in the numbers of *Nitrospirae* strains requiring nitrogen. *Chlorobacteria* and *Nitrospirae* were also reported to be the dominant bacteria in activated sludge in wastewater treatment, which can degrade ammonia-nitrogen and organic chlorides [34–36]. *Acidobacteria* plays an important role in maintaining the pH value [37,38] and influences metal uptake activity [39,40]. *Bacteroidetes* is important for degrading cellulose and chitin [41–44]. On the other hand, morel is usually found growing in forests [45,46], where there are large amounts of fallen leaves that are high in cellulose. Consequently, these afore-mentioned bacteria played different roles for morel growth, and need further research in order to improve the development of morel.

The morel yields in this study were clearly correlated with soil bacterial diversity and richness; these differences were derived from different trace elements or their complexes. There are typically bountiful morel harvests in forests that have been subject to fire, which provides many trace elements via the ashes of trees and grasses [47,48]. Robbins also reported that minerals benefit morel growth [49]. We also measured the contents of five minerals in morel ascocarp and found greater amounts of those elements in experimental groups (except Zn∙Fe∙Mn) than the ck group. In addition, the yields of groups with trace elements were significantly higher than that of the control group (P<0.01), also indicating that trace elements can improve morel yields.

Fe was the largest microelement found in *Morchella*, and its average value (668.38 mg/kg) was similar to that of *Craterellus odoratus* [50]. A variety of studies from European countries have established intake estimates of Se ranging between 38 and 286 μg/day [51]. The average level (118.82 mg/kg) of Se is approximately 9.43 times higher than the amount found in *Russula virescens* [50]. The highest content of Cu in ascocarp in this study was measured at 51.43 mg/kg (group Mn) which is safe for consumption (less than 100 mg/kg) [52]. The contents of Mn and Zn in this study agreed with the report by Ayazs [53]. Though microelements were rich in the artificial morel ascocarp, they still complied with the standards declared by the EU Scientific Committee.

To date, the cultivation of morel still uses semi-artificial planting. This study demonstrated that trace elements affect not only the mycelia and sclerotia growth, the yields and mineral contents of ascocarp, but also bacterial community structures. Based on this research, further studies should focus on determining which types of microbes directly or indirectly affect ascocarp yield. Many similar research achievements and breakthroughs will thrust the morel industry to a new level.

## Supporting information

S1 Fig Mycelia characteristics of morels under a zoom stereomicroscope (16×). The morphology of mycelia showed no obvious difference between the control group and treatment groups. Scale bars represent 100 μm.
(TIF)

S2 Fig Mycelia bridge on medium under stereomicroscope (16 ×). Reticular formation was formed by hyphal branching.
(TIF)

S3 Fig Rarefaction curves (97% similarity). (TIF)

S4 Fig Analyses of principal coordinates using weighted UniFrac. Distance shows that the differences or similarities of soil bacteria community structures among different groups.
(TIF)

S1 Table Diversity indices calculated using a cutoff of 97% similarity. (DOC)
