# Supplementary figures and images for: Effects of element complexes containing Fe, Zn and Mn on artificial morel’s biological characteristics and soil bacterial community structures
Source: PLoS One. 2017 Mar 28;12(3):e0174618. doi: 10.1371/journal.pone.0174618 (PMC5370159; doi:10.1371/journal.pone.0174618)

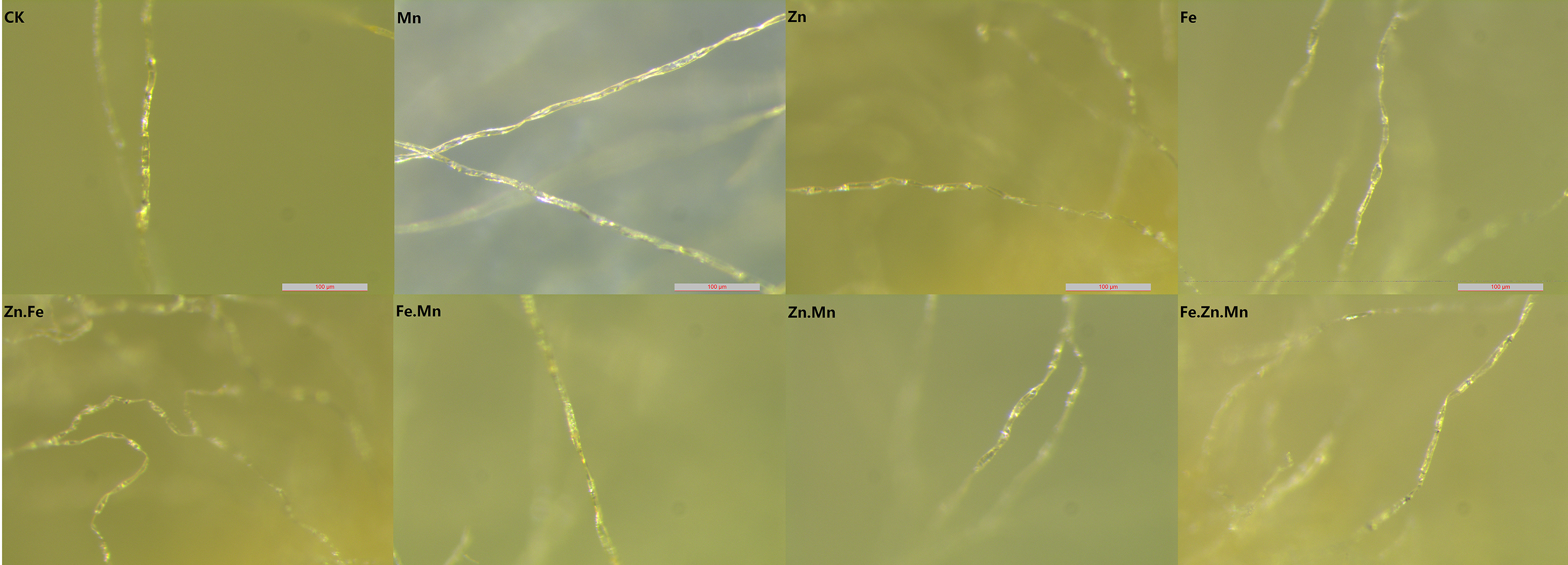

Supplement: S1 Fig — The morphology of mycelia showed no obvious difference between the control group and treatment groups. Scale bars represent 100 μm. (TIF) [file pone.0174618.s001.tif]

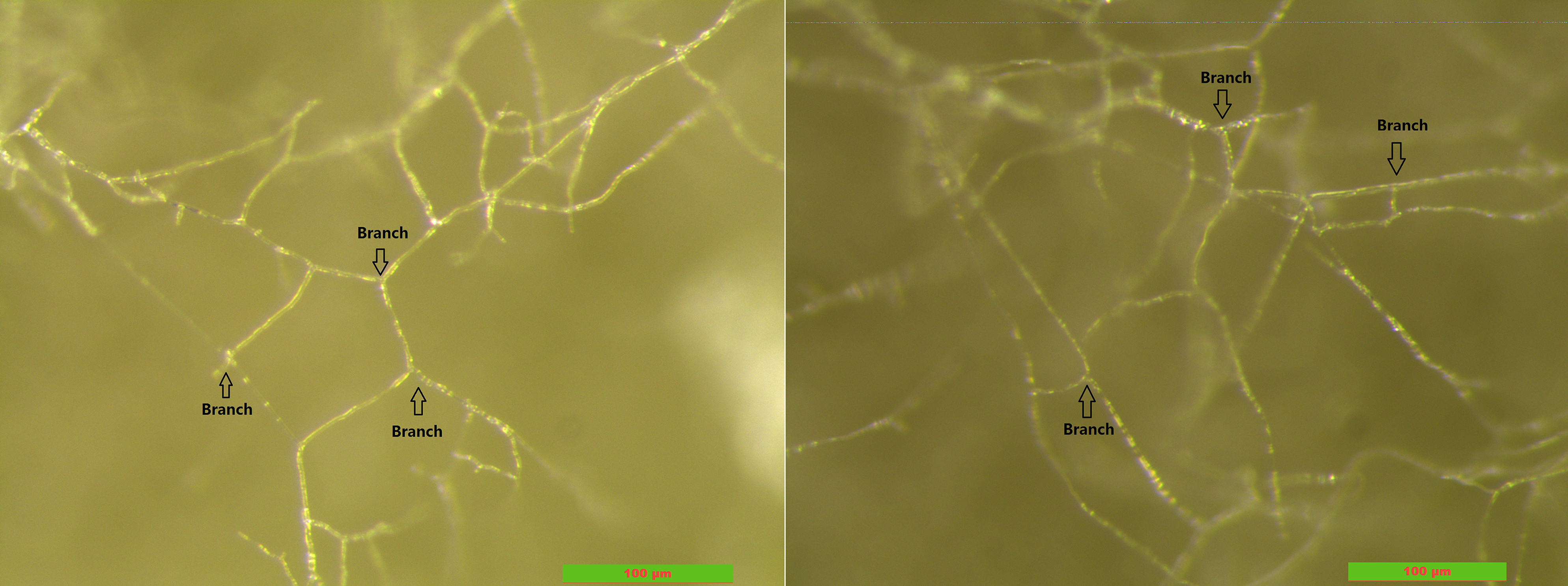

Supplement: S2 Fig — Reticular formation was formed by hyphal branching. (TIF) [file pone.0174618.s002.tif]

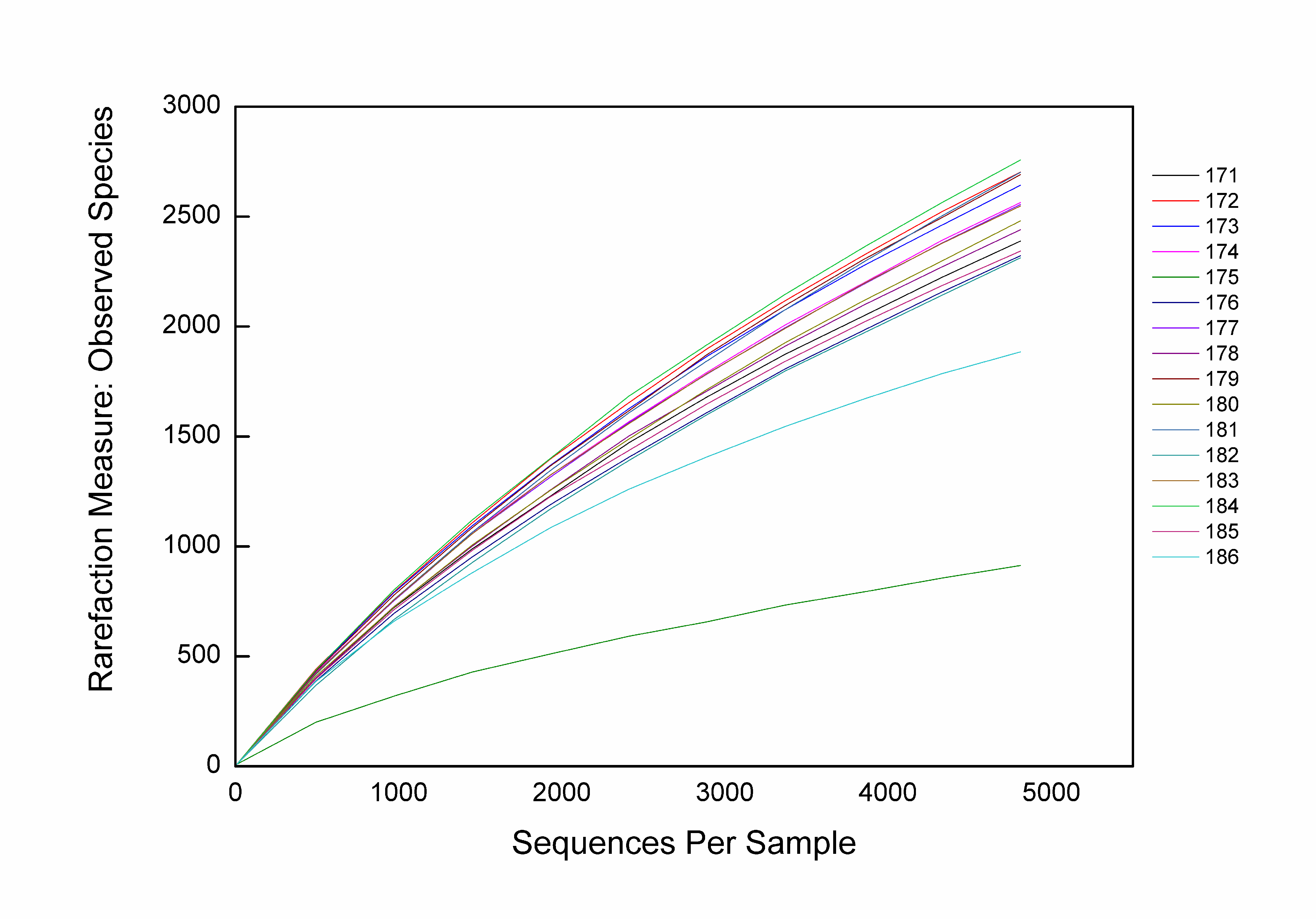

Supplement: S3 Fig — (TIF) [file pone.0174618.s003.tif]

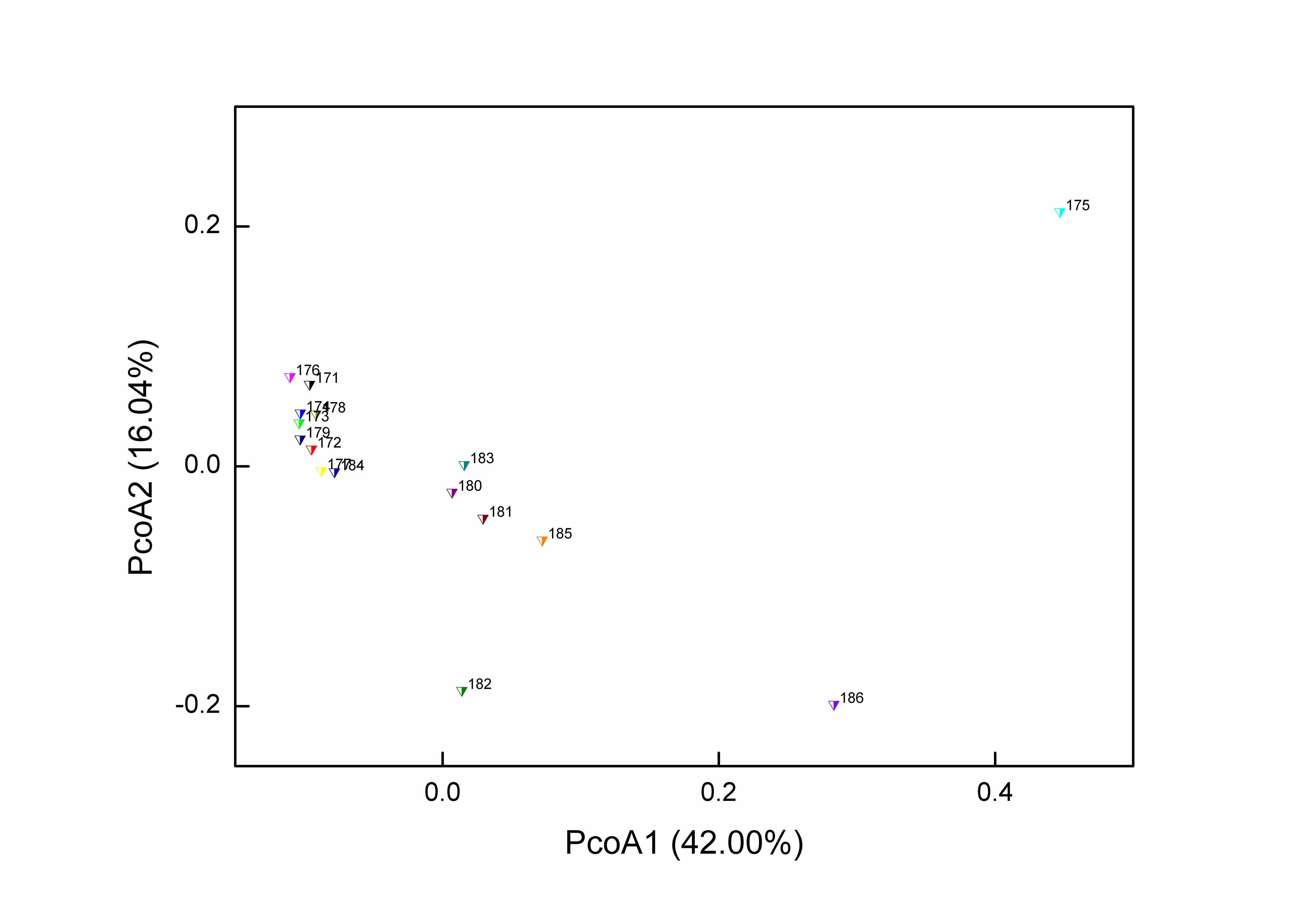

Supplement: S4 Fig — Distance shows that the differences or similarities of soil bacteria community structures among different groups. (TIF) [file pone.0174618.s004.tif]
