# Supplementary material for: Effects of element complexes containing Fe, Zn and Mn on artificial morel’s biological characteristics and soil bacterial community structures
Source: PLoS One. 2017 Mar 28;12(3):e0174618. doi: 10.1371/journal.pone.0174618 (PMC5370159; doi:10.1371/journal.pone.0174618)
Supplement: S1 Table — (DOC) [file pone.0174618.s005.doc]

**S1** Table Diversity indices calculated using a cutoff of 97 % similarity

| Sampled time | Group No. | Chao1 | Observed OTU | Shannon index |
| --- | --- | --- | --- | --- |
| Primordial differentiation stage | 171(ck) | 5774.10 | 2388.10 | 10.53 |
|  | 172(Mn) | 5809.09 | 2702.10 | 10.96 |
|  | 173(Zn) | 5842.02 | 2642.50 | 10.91 |
|  | 174(Fe) | 6259.93 | 2564.00 | 10.78 |
|  | 175(Zn∙Fe) | 2146.03 | 913.40 | 7.65 |
|  | 176(Fe∙Mn) | 6176.06 | 2322.00 | 10.33 |
|  | 177(Zn∙Mn) | 5861.96 | 2555.30 | 10.74 |
|  | 178(Zn∙Fe∙Mn) | 5437.79 | 2440.40 | 10.50 |
| Ascocarp growth stage | 179(ck) | 6383.32 | 2690.90 | 10.88 |
|  | 180(Mn) | 6538.01 | 2480.00 | 10.57 |
|  | 181(Zn) | 7157.31 | 2701.10 | 10.79 |
|  | 182(Fe) | 5980.74 | 2312.50 | 10.05 |
|  | 183(Zn∙Fe) | 5968.72 | 2548.20 | 10.73 |
|  | 184(Fe∙Mn) | 6441.98 | 2756.90 | 11.00 |
|  | 185(Zn∙Mn)) | 5340.15 | 2343.00 | 10.45 |
|  | 186(Zn∙Fe∙Mn) | 3055.63 | 1884.70 | 10.11 |
